# Decolonizing global health: a scoping review of its key components, proposed actions, and contributors

**DOI:** 10.1186/s41256-025-00436-8

**Published:** 2025-10-28

**Authors:** Michelle Amri, Jan Filart, Jinny Yang, Johanna Manga, Kathryn Barrett, Jesse B. Bump

**Affiliations:** 1 The W. Maurice Young Centre for Applied Ethics, School of Population and Public Health, University of British Columbia, 2206 East Mall, Vancouver, BC V6T 1Z3 Canada; 2 Faculty of Health Sciences, Simon Fraser University, 8888 University Dr W, Burnaby, BC V5A 1S6 Canada; 3 Faculty of Arts, McGill University, Dawson Hall, 853 Sherbrooke Street West, Montreal, QC H3A 0G5 Canada; 4 Montreal, Canada; 5 University of Toronto Scarborough Library, 1265 Military Trail, Scarborough, ON M1C 1A4 Canada; 6 Takemi Program in International Health, Harvard T.H. Chan School of Public Health, 665 Huntington Avenue, Bldg. 1, Boston, MA 02115-6021 USA; 7 Bergen Centre for Ethics and Priority Setting, University of Bergen, Bergen, Norway

## Abstract

**Introduction:**

Although there has been attention paid to decolonizing global health, there is no consensus around the concept. To act in the face of various crises, including neocolonialism, there is a need to understand the key components of this concept within mainstream global health, how it can be acted on, and who is contributing to these discussions.

**Methods:**

A scoping review was undertaken to assess the academic literature for discussions on decolonizing global health. The PRISMA guidelines for Scoping Reviews (PRISMA-ScR) were used to guide reporting. OVID Medline, OVID Embase, EBSCO CINAHL Plus, Web of Science Core Collection, PAIS Index, Worldwide Political Science Abstracts, and the International Bibliography of the Social Sciences databases were searched from inception to August 8, 2023. The inclusion criterion was that texts had to: (i) use the exact phrasing of “decoloni* global health” or “anticolonial global health,” (ii) include substantive discussion of what decolonizi* global health or anticolonial global health means (i.e., single mentions that do not include an explanation, elaboration, or context were excluded), and (iii) be published in English.

**Results:**

When analyzing how scholars understand “decolonizing global health”, its meaning is rooted in three key components: (i) power asymmetries between the global north and south; (ii) a legacy of colonialism in global health or neocolonialism; and (iii) epistemic injustice. The second part of the analysis looked to understand if decolonizing global health can be acted on, and if so, how? The analysis demonstrated that decolonization of global health involves: (i) overhauling existing power structures; (ii) establishing agency and self-determination of the global south; (iii) epistemic reformation and epistemic and ontological pluralism; (iv) education; and (v) inclusivity, solidarity, and allyship. Lastly, in assessing which scholars’ work was retrieved in this systematic search of the literature, most first authors were situated in the Americas Region (n = 45/99; 46%), followed by the European Region (n = 29/99; 29%). When combining these two regions, this accounted for almost 75% of all included articles. Notably, only 22% of first authors of retrieved articles had an affiliation in a low- and/or middle-income country.

**Conclusions:**

The findings from this scoping review are anticipated to bring much needed clarity to discussions around decolonizing global health, in terms of key components, gaps, and possible actions. For instance, this review presents ongoing challenges faced in coming to a comprehensive and agreed upon definition of decolonizing global health in mainstream global health.

## Introduction

There has long been recognition of the pervasiveness of colonization. In 1965, Kwame Nkrumah wrote about the continued exercise of control, mainly by economic means, even after political decolonization [1]. More recently, the concept of neocolonialism has been invoked to describe “the present-day consequences of colonialism and/or various ongoing manifestations of the power inequalities of that period” [2]. Within global health, there is growing recognition that the field cannot continue to operate in its current form to achieve its sought out aims, particularly those focused on health equity, without drastic changes.

Despite increasing attention to ‘decolonizing global health’ [3, 4], there is no consensus on what it means [5]. For instance, the *Global Health Research and Policy* Symposium in 2021 reached consensus that to “fully decolonize global health, systemic reforms must be taken that target the fundamental assumptions of global health: does investment in global health bring socioeconomic development, or is it the other way around?” [6]. Per the symposium, decolonizing global health requires targeting fundamental assumptions. Büyüm et al. note that “Decolonising global health advances an agenda of repoliticising and rehistoricising health through a paradigm shift, a leadership shift and a knowledge shift” [7]. Their approach centers around a broader shift in thinking and practice. Abimbola and Pai note that “to decolonise global health is to remove all forms of supremacy within all spaces of global health practice, within countries, between countries, and at the global level” [8]. Their approach focuses on eliminating supremacy. Despite these efforts, discussions around addressing decolonization widely vary [9], and what “decolonizing global health” entails remains diverse and not solidified [1].

This present study contributes to the growing decolonization of global health literature and related efforts through a scoping review in accordance with the protocol [1]. In this article, the following research question was answered: “what does the literature say about decolonizing global health—what does it mean and how should actors best proceed?” [1]. Who was participating in these mainstream global health discussions was also examined [1]. This study sought to provide clarity around decolonizing global health, in terms of key components in its meaning(s), gaps, and possible actions [1]. The focus was on providing deep conceptual clarity over comprehensive actionable solutions, an area in which other newly available work excels [10]. Conceptual clarity can ensure mutual understanding among stakeholders, promote critical refection, prompt reconsideration of assumed understandings, and has the potential to unveil aspects previously unconsidered.

## Methods

The present study compiled findings from the academic literature in line with the scoping review method, whereby a scoping review “aims to identify the nature and extent of research evidence” [11]. This work focused on better understanding what decolonizing global health means within mainstream global health databases and how it can be acted on [1]. The PRISMA guidelines for Scoping Reviews (PRISMA-ScR) were followed to guide reporting [12], as per the published protocol [1].

### Eligibility criteria

The inclusion criteria were that texts had to: (i) use the exact phrasing of “decoloni* global health” or “anticolonial global health,” (ii) include substantive discussion of what decolonizi* global health or anticolonial global health means, and (iii) be published in English [1]. Whereas the exclusion criteria included: (i) texts that do not substantively discuss decolonizing global health (i.e., single mentions without explanations, elaboration, or context were excluded) and (ii) grey literature articles [1].

### Information sources and search strings

**Fig. 1:**
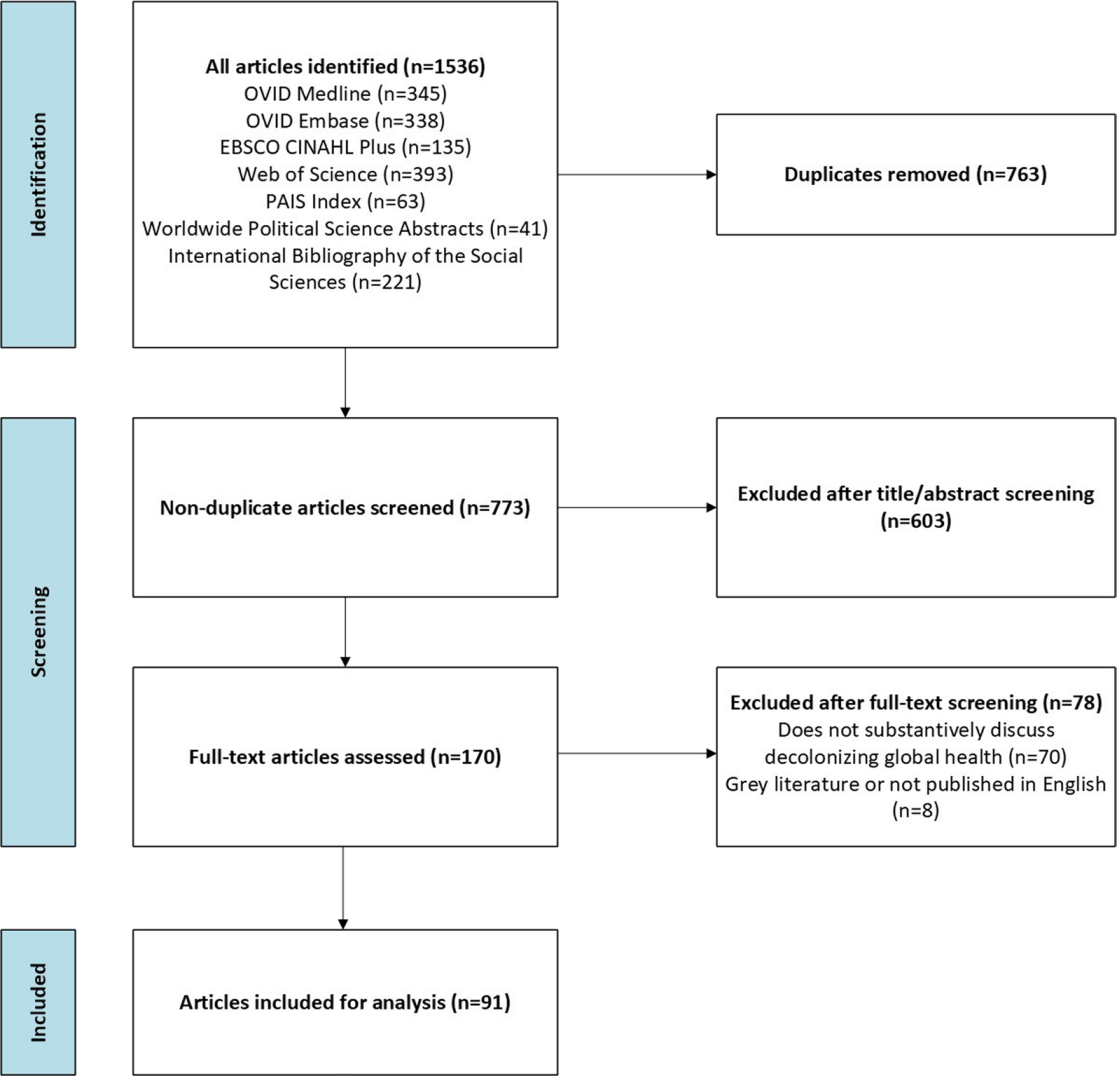
PRISMA flow diagram

A robust search strategy was crafted in collaboration with an academic librarian (KB) and searched for articles in OVID Medline, OVID Embase, EBSCO CINAHL Plus, Web of Science Core Collection (Arts & Humanities Citation Index, Science Citation Index Expanded, Social Sciences Citation Index, Conference Proceedings Citation Index—Science, Conference Proceedings Citation Index—Social Sciences and Humanities, Emerging Sources Citation Index), ProQuest PAIS Index, ProQuest Worldwide Political Science Abstracts, and the ProQuest International Bibliography of the Social Sciences databases from inception to August 8, 2023 (when the search was conducted). No limits were applied to the searches. The rationale for searching each database and search strings are available in the published protocol [1]. The number of results from each database searched is indicated in the PRISMA flow diagram (Fig. 1).

### Data selection and collection process

Two stages of screening were undertaken. In the first stage, each title and abstract was read by two independent reviewers to determine inclusion in the second stage. During the second stage, full texts were read by two independent reviewers to determine alignment with the inclusion criteria and to confirm they did not meet the exclusion criteria. Conflicts were resolved in a consultative manner and all articles included in this study were approved by the full authorship team. The PRISMA flow diagram shows each screening stage and associated article exclusions (Fig. 1). All articles included in the study are presented in Table 1.

**Table 1:** Bibliographic information for included articles

Reference	Author(s)	First author’s institution(s)	Year	Article title	Journal
[5]	Krugman	Department of International Health, Johns Hopkins Bloomberg School of Public Health, Baltimore, Maryland, United States of America	2023	Global health and the elite capture of decolonization: On reformism and the possibilities of alternate paths	PLOS Global Public Health
[6]	Kwete et al	Global Health Research and Consulting, Yaozhi, Yangzhou, China	2022	Decolonizing global health: what should be the target of this movement and where does it lead us?	Global Health Research and Policy
[7]	Büyüm et al	Duke Global Health Institute, Durham, North Carolina, USA	2020	Decolonising global health: if not now, when?	BMJ Global Health
[8]	Abimbola & Pai	School of Public Health, University of Sydney, Sydney, Australia	2020	Will global health survive its decolonisation?	The Lancet
[9]	Finkel et al	Weill Cornell Medicine, New York, NY, USA	2022	What Do Global Health Practitioners Think about Decolonizing Global Health?	Annals of Global Health
[13]	Besson	Department of Infectious Diseases Epidemiology, London School of Hygiene & Tropical Medicine, London WC1E 7HT, UK	2021	Confronting whiteness and decolonising global health institutions	The Lancet
[14]	Ferron Guzman & Rowthorn	The Graduate School, University of Maryland, Baltimore, MD	2022	Introduction to Special Collection on Decolonizing Education in Global Health	Annals of Global Health
[15]	Abimbola et al	School of Public Health, University of Sydney, Sydney, Australia	2021	Addressing power asymmetries in global health: Imperatives in the wake of the COVID-19 pandemic	PLOS Medicine
[16]	Hellowell & Schwerdtle	Global Health Policy Unit, School of Social and Political Science, University of Edinburgh, Edinburgh, UK	2021	Powerful ideas? Decolonisation and the future of global health	BMJ Global Health
[17]	Kunnuji et al	Department of Sociology, University of Lagos, Akoka, Lagos, Nigeria	2023	Why ‘elevating country voice’ is not decolonizing global health: A frame analysis of in-depth interviews	PLOS Global Public Health
[18]	Sekalala et al	Warwick Law School, University of Warwick, Coventry, UK	2021	Decolonising human rights: how intellectual property laws result in unequal access to the COVID-19 vaccine	BMJ Global Health
[19]	Stevens-Uninsky et al	Department of Global Health, McMaster University, Hamilton, ON, Canada	2023	Decolonization in Sexual and Reproductive Health Research Methods: Protocol for a Scoping Review	JMIR Research Protocols
[20]	Renmans et al	Institute of Development Policy, University of Antwerp, Antwerpen, Belgium	2022	Realist evaluation in times of decolonising global health	The International Journal of Health Planning and Management
[21]	Chigudu	Oxford Department of International Development, University of Oxford	2021	An ironic guide to colonialism and global health	The Lancet
[22]	Hussain et al	Ross University School of Medicine, Miramar, FL, USA	2023	Colonization and decolonization of global health: which way forward?	Global Health Action
[23]	Tang et al	Acacia Lab for Implementation Science, School of Health Management and Dermatology Hospital, Southern Medical University, Guangzhou, China	2023	The implications of decolonization on China’s academic global health: a dialogue with Stephen Gloyd at the Luhu Global Health Salon	Global Health Research and Policy
[24]	Harper & Pratt	School of Population and Global Health, University of Melbourne, Australia	2021	Combatting neo-Colonialism in Health Research: What can Aboriginal Health Research Ethics and Global Health Research Ethics Teach Each Other?	Journal of Empirical Research on Human Research Ethics
[25]	Daffé et al	Center for Global Health, Massachusetts General Hospital, Boston, Massachusetts	2021	Anti-Racism and Anti-Colonialism Praxis in Global Health-Reflection and Action for Practitioners in US Academic Medical Centers	The American journal of tropical medicine and hygiene
[26]	Hindmarch & Hillier	Department of Political Science, University of New Brunswick, Fredericton, Canada	2023	Reimagining global health: From decolonisation to indigenization	Global Public Health
[27]	Jock et al	Centre for Indigenous Peoples’ Nutrition & Environment (CINE), McGill University, Montreal, Canada	2021	Dismantling the status quo: promoting policies for health, well-being and equity: an IUHPE2022 prelude	Global Health Promotion
[28]	Binagwaho et al	University of Global Health Equity, RW	2022	Eliminating the White Supremacy Mindset from Global Health Education	Annals of Global Health
[29]	Polidano et al	School of Medicine, Keele University, Newcastle-under-Lyme, United Kingdom,	2022	Community Engagement in Cutaneous Leishmaniasis Research in Brazil, Ethiopia, and Sri Lanka: A Decolonial Approach for Global Health	Frontiers in Public Health
[30]	Pratt & de Vries	Queensland Bioethics Centre, Australian Catholic University—Brisbane Campus, Banyo, Queensland, Australia	2022	Where is knowledge from the global South? An account of epistemic justice for a global bioethics	Journal of Medical Ethics
[31]	Adhikari et al		2023	The way forward in decolonising global health	The Lancet Global Health
[32]	Bua & Sahi	Department of Surgery, Busitema University Faculty of Health Sciences, Mbale, Uganda	2022	Decolonizing the decolonization movement in global health: A perspective from global surgery	Frontiers in Education
[33]	Guillaume et al	Center for Infectious Disease and Nursing Innovation, Johns Hopkins University, Baltimore, MD, USA; Jhpiego, Baltimore, MD, USA	2023	Decolonization of Global Health in Haiti: A Call for Equity, Partnerships, Scholarship, and Informed Action	Global Health: Science and Practice
[34]	Hirsch	London School of Hygiene and Tropical Medicine	2021	Is it possible to decolonise global health institutions?	The Lancet
[35]	Delormier et al. *†		2022	Tiohtià:ke Statement: Catalysing policies for health, well-being and equity	Global Health Promotion
[36]	Forsberg & Sundewall	Department of Global Public Health, Karolinska Institutet, Stockholm, Sweden	2023	Decolonizing global health—what does it mean for us?	European Journal of Public Health
[37]	Bump & Aniebo	Department of Global Health, Harvard T.H. Chan School of Public Health, Boston, MA; Initiative on the Future of Health and Economic Resiliency in Africa, Boston, MA; Bergen Center for Ethics and Priority Setting, University of Bergen, Bergen, Norway	2022	Colonialism, malaria, and the decolonization of global health	PLOS Global Health
[38]	Qato	University of Maryland Baltimore, School of Medicine & School of Pharmacy, US	2022	Reflections on ‘Decolonizing’ Big Data in Global Health	Annals of Global Health
[39]	Abimbola & Pai	School of Public Health, University of Sydney, Sydney, Australia	2021	Author reply: Undoing supremacy in global health will require more than decolonisation	The Lancet
[40]	Chawla et al	Médecins Sans Frontières Amsterdam, Holland and International Committee of Red Cross Geneva, Geneva, Switzerland	2022	Post-decolonisation: Global Health and Global Surgery’s Coming of Age	Indian Journal of Surgery
[41]	Mtima-Jere et al	Department of Rehabilitation Sciences, Kamuzu University of Health Sciences, School of Life Sciences and Allied Health Professions, Blantyre, Malawi	2023	Understanding colonialism and its influences on contemporary physiotherapy education and research: students’ perspectives on decolonializing solutions	Physiotherapy Theory and Practice
[42]	Rees et al	Division of Pediatric Emergency Medicine, Emory University School of Medicine, Atlanta, GA	2023	Has Authorship in the Decolonizing Global Health Movement Been Colonized?	Annals of Global Health
[43]	Affun-Adegbulu & Adegbulu	Department of Public Health, Institute of Tropical Medicine, Antwerpen, Belgium; Department of Political Sciences, University of Antwerp, Antwerpen, Belgium	2020	Decolonising Global (Public) Health: from Western universalism to Global pluriversalities	BMJ Global Health
[44]	Perkins et al	Center for Indigenous Health, Johns Hopkins University Bloomberg School of Public Health, Baltimore, Maryland, USA	2023	Educational approaches to teach students to address colonialism in global health: a scoping review	BMJ Global Health
[45]	Lawrence & Hirsch	Department of Clinical Research, Faculty of Infectious and Tropical Diseases, The London School of Hygiene and Tropical Medicine, London, UK; Botswana Harvard AIDS Institute Partnership, Gaborone, Botswana	2020	Decolonising global health: transnational research partnerships under the spotlight	International Health
[46]	Demir	School of Sociology and Social Policy, University of Leeds, LS2 9JT, UK	2022	How and Why Should We Decolonize Global Health Education and Research?	Annals of Global Health
[47]	Oti & Ncayiyana	The Secretariat, Network of Impact Evaluation Researchers in Africa, Nairobi, Kenya; Panel of Movers, Global Health Decolonisation Movement in Africa, Nairobi, Kenya	2021	Decolonising global health: where are the Southern voices?	BMJ Global Health
[48]	Sridhar et al	Division of General Internal Medicine and Primary Care, Brigham and Women’s Hospital, Boston, MA, USA	2023	Learning to walk the walk: Incorporating praxis for decolonization in global health education	Global Public Health
[49]	Khan et al	Faculty of Public Health and Policy, London School of Hygiene and Tropical Medicine, London, UK	2021	Decolonising global health in 2021: a roadmap to move from rhetoric to reform	BMJ Global Health
[50]	Foretia	Division of Health Policy and Research, Nkafu Policy Institute, Yaoundé, Cameroon; Center for Multicultural and Global Health, University of Tennessee Health Science Center, Memphis, TN, USA; Global Surgery Institute, University of Tennessee Health Science Center, Memphis, TN, USA	2022	To decolonize global surgery and global health we must be radically intentional	The American Journal of Surgery
[51]	Olusanya et al	Centre for Healthy Start Initiative, Lagos, Nigeria	2021	Transforming global health through equity-driven funding	Nature Medicine
[52]	Piñones-Rivera et al	Department of Social Sciences at the Universidad de Tarapacá, Iquique, Chile	2023	Global Social Medicine for an Equitable and Just Future	Health and Human Rights Journal
[53]	Sen et al	Wolfson College (CR), University of Oxford	2022	Understanding the Context of Global Health Policies: Their Post-Colonial Legacies and Impacts on Health Service Systems	World Review of Political Economy
[54]	Contractor & Dasgupta	Department of Public Health, Institute of Tropical Medicine, Antwerp, Belgium; The George Institute for Global Health India, New Delhi, India; Centre for International Health, Department of Global Public Health & Primary Care, University of Bergen, Bergen, Norway	2022	Is decolonisation sufficient?	BMJ Global Health
[55]	Keshri & Bhaumik	The George Institute for Global Health, Faculty of Medicine and Health, University of New South Wales, Sydney, New South Wales, Australia; Injury Division, The George Institute for Global Health India, New Delhi, Delhi, India	2022	The feudal structure of global health and its implications for decolonisation	BMJ Global Health
[56]	Khan & Shanks	Department of Global Health and Development, London School of Hygiene & Tropical Medicine, London, UK; Community Health Sciences, Aga Khan University, Pakistan	2020	Decolonising COVID-19: delaying external debt repayments	The Lancet Global Health
[57]	Rivera-Segarra et al.* §*	School of Behavioral and Brain Sciences, Ponce Health Sciences University, Ponce, Puerto Rico	2022	Global mental health research and practice: a decolonial approach	Lancet Psychiatry
[58]	Ssennyonjo et al	Department of Health Policy Planning and Management, School of Public Health, Makerere University, Kampala, Uganda; Institute of Development Policy, University of Antwerp, Antwerp, Belgium; Department of Public Health, Institute of Tropical Medicine (ITM), Antwerp, Belgium	2023	The ‘decolonization of global health’ agenda in Africa: harnessing synergies with the continent’s strategic aspirations	European Journal of Public Health
[59]	Arguedas-Ramírez	School of Philosophy, Universidad de Costa Rica, San Jose, Costa Rica	2021	Build that wall! Vaccine certificates, passes and passports, the distribution of harms and decolonial global health justice	Journal of Global Ethics
[60]	Barthelemy et al	Global Neurosurgery Laboratory, Division of Neurosurgery, Department of Surgery, SUNY Downstate Health Sciences University, Brooklyn, NY; Society of Haitian Neuroscientists, Inc., New York, NY	2023	Historical determinants of neurosurgical inequities in Africa and the African diaspora: A review and analysis of coloniality	PLOS Global Public Health
[61]	Besson	Department of Infectious Disease Epidemiology, London School of Hygiene and Tropical Medicine, London, UK	2022	How to identify epistemic injustice in global health research funding practices: a decolonial guide	BMJ Global Health
[62]	Chapman et al	Department of Anthropology, University of Washington, Seattle, WA, USA	2022	Decolonising the global to local movement: Time for a new paradigm	Global Public Health
[63]	Chaudhuri et al	Orillia Soldiers’ Memorial Hospital, Orillia, Ontario, Canada	2021	Decolonising global health: beyond ‘reformative’ roadmaps and towards decolonial thought	BMJ Global Health
[64]	Decamp et al	Fulginiti Pavilion for Bioethics and Humanities, Aurora, CO	2023	Decolonizing Global Health Research: Perspectives from US and International Global Health Trainees	Annals of Global Health
[65]	Dee & Lasco	Department of Radiation Oncology, Memorial Sloan Kettering Cancer Center, New York, NY	2022	Decolonising global health: a Philippine perspective	The Lancet
[66]	Eichbaum et al	Vanderbilt University Medical Center, Nashville, TN	2021	Decolonizing Global Health Education: Rethinking Institutional Partnerships and Approaches	Academic medicine: Journal of the Association of American Medical Colleges
[67]	Fofana	Harvard-Affiliated Emergency Medicine Residency (HAEMR), Boston, MA, USA	2020	Decolonising global health in the time of COVID-19	Global Public Health
[68]	Gallego-Perez	Department of Physical Medicine and Rehabilitation, University of North Carolina at Chapel Hill, Chapel Hill, NC, USA	2023	Characterizing Therapeutic Pluralism Policies in Latin America: A Qualitative Content Analysis	Journal of Integrative and Complementary Medicine
[69]	Garba et al.* ††*	Program in Global Surgery and Social Change, Harvard Medical School, Boston MA, US; University of North Carolina School of Medicine, Chapel Hill, NC, US	2021	How Do We Decolonize Global Health in Medical Education?	Annals of Global Health
[70]	Gautier et al	Département de Gestion, Évaluation et Politique de Santé, Université de Montréal, Montreal, Canada; Centre de recherche en santé publique, Université de Montréal et CIUSSS du Centre-Sud-de-l’Île-de-Montréal, Montreal, Canada; Department of Sociology, McGill University, Montreal, Canada	2020	Rethinking development interventions through the lens of decoloniality in sub-Saharan Africa: The case of global health	Global Public Health
[71]	Herrick & Bell	Department of Geography, King’s College London, London, UK	2022	Epidemic confusions: On irony and decolonisation in global health	Global Public Health
[72]	Herrick et al	Department of Geography King’s College London, London, WC2R 2LS, UK	2021	Unequal ecosystems of global health authorial expertise: Decolonising noncommunicable disease	Health & Place
[73]	Hirsch	Department of Geography, University College London, London, UK	2019	In the wake: Interpreting care and global health through Black geographies	Area
[74]	Hommes et al	London School of Hygiene & Tropical Medicine, London WC1E 7HT, UK	2021	The words we choose matter: recognising the importance of language in decolonising global health	The Lancet Global Health
[75]	Horton		2021	Offline: The real meaning of decolonisation	The Lancet
[76]	Iwelunmor	Behavioral Science and Health Education, Saint Louis University, Saint Louis, MO	2022	The truth about decolonising global health worth spreading	The Lancet
[77]	Jantsch	Secretaria Executiva da UNA-SUS (SE/UNA-SUS), Universidade Aberta do SUS, Brasılia, Brazil	2023	Decolonizing global health—what is the plan?	European Journal of Public Health
[78]	Jensen & Lopez-Carmen	Oglala Lakota Nation, Oceti Sakowin Land, United States of America; School of Engineering, Stanford University, Stanford, California, United States of America	2022	The “Elephants in the Room” in U.S. global health: Indigenous nations and white settler colonialism	PLOS Global Public Health
[79]	Kalbarczyk et al	International Health, Johns Hopkins University Bloomberg School of Public Health, Baltimore, Maryland, USA	2023	Using antioppressive teaching principles to transform a graduate global health course at Johns Hopkins University	BMJ Global Health
[80]	Keikelame & Swartz	Department of Psychology, Stellenbosch University, Cape Town, South Africa	2019	Decolonising research methodologies: lessons from a qualitative research project, Cape Town, South Africa	Global Health Action
[81]	Khan	Department of Surgery, Independent Medical College, Faisalabad, Pakistan	2022	Decolonising global health by decolonising academic publishing	BMJ Global Health
[82]	Kulesa & Brantuo	Graduate School of Education and Human Development, George Washington University, Washington, DC, USA; Department of Hospital Medicine, Children’s National Hospital, Washington, DC, USA	2021	Barriers to decolonising educational partnerships in global health	BMJ Global Health
[83]	Kulesa et al	Department of Pediatrics, George Washington University School of Medicine and Health Sciences, Washington, DC; Division of Hospital Medicine, Children’s National Hospital, Washington, DC	2022	Prioritization and Resource Allocation in Academic Global Health Partnerships	Academic Pediatrics
[84]	Lokugamage	Medical School, University College London, London WC1E 6DE, UK	2021	Transformational learning to decolonise global health	The Lancet
[85]	MacDonald et al	Department of Paediatrics, Dalhousie University, IWK Health Centre, Halifax, Nova Scotia, Canada	2022	A long-term process for decolonizing and democratizing community-focused research: the case for MicroResearch in East Africa and in Canada	Canadian Journal of Public Health
[86]	Mika	Center for Science, Technology, Medicine, and Society, University of California, Berkeley, USA	2022	From Africanizing oncology to decolonizing global health: reflections on the biomedical turn in African health histories	Africa: The Journal of the International African Institute
[87]	Mogaka et al	Kenya Medical Research Institute, Nairobi, Kenya	2021	Why and for whom are we decolonising global health?	The Lancet Global Health
[88]	Newman	Department of Conflict and Development Studies, Ghent University, Ghent, Belgium	2023	Decolonising social norms change: from ‘grandmother-exclusionary bias’ to ‘grandmother-inclusive’ approaches	Third World Quarterly
[89]	Olusanya	Centre for Healthy Start Initiative, Lagos, Nigeria	2021	Dismantling Structural Discrimination in Global Health	JAMA Pediatrics
[90]	Pandhi	Princeton University	2022	Review: Richardson’s Epidemic Illusions	Journal of the Royal Anthropological Institute
[91]	Pant et al	Department of Prevention and Community Health, George Washington University School of Public Health, Washington, DC	2022	Decolonising global health evaluation: Synthesis from a scoping review	PLOS Global Public Health
[92]	Passos et al	Abraço a microcefalia, Salvador, Brazil	2020	The promise and pitfalls of social science research in an emergency: lessons from studying the Zika epidemic in Brazil, 2015–2016	BMJ Global Health
[93]	Prasad et al	University of Minnesota	2022	Global Health Partnerships and the Brocher Declaration: Principles for Ethical Short-Term Engagements in Global Health	Annals of Global Health
[94]	Rasheed	Department of Paediatrics and Child Health, The Aga Khan University, Karachi, Pakistan	2021	Navigating the violent process of decolonisation in global health research: a guideline	The Lancet Global Health
[95]	Ratner et al	Division of Respiratory Medicine, Boston Children’s Hospital, Boston, MA, US	2022	Learner Milestones to Guide Decolonial Global Health Education	Annals of Global Health
[96]	Smith et al	Department of Geography and Planning, The University of Liverpool, Liverpool, UK	2021	Desire over damage: Epistemological shifts and anticolonial praxis from an indigenous-led community health project	Sociology of Health & Illness
[97]	Somerville & Munguambe	Gender Centre and Fellow of the Global Health Centre, Graduate Institute of International and Development Studies, Geneva, Switzerland	2021	The rise of non-communicable disease (NCDs) in Mozambique: decolonising gender and global health	Gender & Development
[98]	Tilley	History Department, Northwestern University, Evanston, IL, USA	2021	Traditional Medicine Goes Global: Pan-African Precedents, Cultural Decolonization, and Cold War Rights/Properties	Osiris

The final articles included in this study were imported into NVivo and coded for the questions of interest: (i) what decolonizing global health means and (ii) how it can be acted on. These findings were synthesized and are presented below.

## Results

A total of 1536 articles were retrieved for screening in this scoping review when the search was conducted across all databases of interest on August 8, 2023. A total of 763 duplicates were either removed automatically in Covidence or manually. In total, 773 article titles and abstracts were screened and 170 full texts were assessed for eligibility. This full-text screening resulted in 78 articles being excluded, 70 of which met the exclusion criteria of not substantively discussing decolonizing global health. Overall, 91 articles were included in this scoping review.

### PART I—What does decolonizing global health mean and what are its key concepts?

Several authors have provided their definitions of what decolonizing global health is. For example, Abimbola and Pai believe it is a removal of all forms of supremacy within global health practice [8]. Whereas Besson shares a more comprehensive yet succinct definition:

*Decolonising global health is a movement to establish our agency and protect our indelible right to participate in scientific advancement on our terms, proudly embracing our cultures. It will only be possible if everyone—especially white people—can “free” ourselves from the idea of the innate superiority of western culture and research paradigms.* [13]

Besson’s definition points to the need for global health to be freed of any form of Western^1^ superiority, while empowering the marginalized by legitimizing non-Western knowledge and cultures. Building on these select definitions, this study sought to investigate what decolonizing global health means to various scholars, both in terms of its definition and key concepts. As such, it is fundamental to elucidate how scholars have critiqued the foundation of the current state of global health. Three key themes in line with this interrogation were uncovered around: (i) power asymmetries between the global north and south, (ii) the legacy of colonialism in global health or neocolonialism, and (iii) epistemic injustice.

#### Theme 1: Power asymmetries between the global north and south

One of the most prominent themes found was highlighting the asymmetry of power between the global north and the global south, and how this shapes the current landscape of global health. Faerron Guzman and Rowthorn break down this asymmetry even further:

*Despite advances in multiple aspects of the decolonization agenda, the entrenched nature of the power asymmetries within global health is still represented by ongoing imbalances in whose voices count, what systems of knowledge are valued, who gets to set the priorities, and who holds the financial resources.* [14]

This quote highlights the various ways in which high-income countries (HICs) continue to dominate the global health landscape despite traction around decolonizing global health. Abimbola et al. draw on the Global Health 50/50 report of 2020 to point out that most global health organizations are headquartered in HICs and are mostly led by men living in and/or educated in HICs [100] as cited by [15]. Scholars felt that the center of global health will continue to be in the global north because its primary funding comes from rich countries [16]. In Kunnuji et al.’s study, one respondent mentioned how HIC donors tend to set their own agendas in doing research in low- and middle-income countries (LMICs) without consulting the recipient country with what their needs or priorities are [17].

Perhaps the most visible power asymmetries in global health were seen during the COVID-19 pandemic, particularly when the vaccine apartheid emerged. Sekalala et al. note the disparities in vaccination that left many LMIC citizens unable to access vaccines despite citizens in richer countries having access [18]. It was further argued how the creation of vaccine passports severely restricted equal freedom of movement among the global population [18].

Ultimately, power imbalances are the result of structures that have been formed to perpetuate HIC dominance of the global health landscape [14]. Most global health research funding comes from HICs, who are the real beneficiaries of projects, at the expense of LMICs [16, 17]. The north consolidates its power by keeping the global south from having any significant influence in global health, as seen from intellectual property laws preventing global south countries from manufacturing COVID-19 vaccines [18].

#### Theme 2: Legacy of colonialism in global health or neocolonialism

Power asymmetries between the global north and south are traced back to the legacies of colonialism. Western hegemony has dominated various aspects of global health, including research. Indigenous knowledge and languages have been marginalized by Western research paradigms such as positivism and constructivism [19]. Renmans et al. suggest an implied hierarchy of knowledge, which holds Western practices as superior, thus disregarding “situated knowledge and indigenous understandings” [20]. In a review of Eugene Richardson’s *Epidemic Illusions*, Chigudu mentions that global public health’s efforts to mold the world in its own image subverts experiences and knowledge of global health’s beneficiaries [21]. It could even be argued that global health is currently a form of neocolonialism, such as intellectual property rights for COVID-19 vaccines being consolidated from countries in the global south [18]. Taking this illustration from Finkel et al., one could see how today’s exploitation of LMICs by HICs in health research appears very similar to the essence of colonial practice:

*The HIC institution generally sets the research agenda, formulates the research questions, designs the study, obtains the funding, retains most of the overheads, conducts the analyses, presents the findings at conferences, and publishes the findings in English in journals that may be unavailable and/or unaffordable to their partner in the LMIC where the study is actually conducted.* [9]

The perpetuation of these practices has led to saviourism and parachute research. When HIC research funders impose their own agendas and act as “saviours” on LMICs, resources are ultimately wasted, and mistrust is fostered [15]. Hussain et al. suggest that HIC researchers travelling to LMICs perpetuate a colonial mentality, as visiting researchers are not often encouraged to investigate root causes of inequities [22]. This type of parachute research tends to be unilateral in terms of knowledge exchange, when it is equally important for those in the host country to share their own knowledge as well. When local knowledge is disregarded, external researchers tend to leave the research site without any local input on findings, and with no credit given to local researchers and experts [20]. As suggested in the Kunnuji et al. study, “he who has the dollar holds the power” [17], meaning that HIC funders not only get to decide how and where to allocate funds, but also get control over data and knowledge ownership, as well as publication of said research [23]. This in turn fosters an LMIC dependence on HICs due to resource inequities. Not enough budget is allocated for investing in better clinical, laboratory, and human resource infrastructures in host countries, which then perpetuate inequities [9].

Evidently, global health’s roots are colonial and its legacies are still present today. The practice of parachute research allows HICs to exploit LMIC resources [9, 15], which resembles colonialism of the past.

#### Theme 3: Epistemic injustice

One concrete definition of epistemic injustice in the context of global health research was suggested in an article by Harper and Pratt as LMIC researchers not receiving respect for their knowledge [24]. Scholars across the articles included in this study noted that both LMIC and Indigenous knowledge are marginalized [25–27]. Daffé et al. point to a framing of these knowledge systems as uncivilized and illegitimate as a way to make it seem that colonial ways are superior [25]. Binagwaho et al. traced the roots of this “colonial epistemicide” to Western hegemony and eurocentric thinking [28]. Polidano et al. point out how Western and eurocentric thinking has established a “hegemonic knowledge hierarchy” such that their perspectives are treated as the only legitimate base for global health research [29]. Besson also quotes Richard Dyer in claiming how Western institutions have “colonized the definition of normal” [13]. The assumption of Western medical knowledge as superior disallows and marginalizes the integration of alternative knowledge and healing practices into the health systems of different countries [16, 17].

Academic publishing and knowledge exchange are HIC-centered. Pratt and de Vries believe that for LMIC scholars to have a better chance at publishing in international journals, they should write from a northern epistemic position, meaning framing their work in reference to global north cultures and knowledge [30]. Furthermore, since the majority of global north-based publications are in English, knowledge produced by non-English speakers is disregarded, and in a way, gatekeeping also occurs [28]. This has fueled the international brain drain of health workers. Adhikari et al. argue that this is a form of modern colonialism, with HICs improving their health systems by capitalizing on labour from LMICs, leaving countries in the global south with labour shortages [31]. Furthermore, Bua and Sahi note that the centralization of academic medical centers in the urban areas of most LMICs cause a domestic brain drain, further marginalizing rural communities as part of healthcare systems [32].

Because of power asymmetries and colonial legacies, global health has delegitimized knowledges from the global south and Indigenous populations [19, 29]. Western medical approaches are widely considered superior even by those from the global south [25], and this results in a brain drain [31] that further allows the global north’s consolidation of power within minds.

### PART II—Can decolonizing global health be acted on? If so, how?

After establishing the key colonial characteristics and legacies of the current global health landscape, this next set of themes compiles authors’ views about how global health can be decolonized. These views were compiled without conducting an evidence appraisal, meaning that there may not be implementation research to support each action, particularly for actions that are inherently more difficult to evaluate. This was intentionally done to recognize that all views provide an important conceptual contribution. The decolonization of global health involved: (i) overhauling existing power structures; (ii) establishing agency and self-determination of the global south; (iii) epistemic reformation and epistemic and ontological pluralism; (iv) education; and (v) inclusivity, solidarity, and allyship.

#### Theme 1: Overhaul existing power structures

It is widely suggested by authors of included articles that power imbalances require an overhaul of existing structures in global health. Because of global health’s colonial legacies, Guillaume et al. imagine that global health would not be the same if its existing power structures were removed:

*These power structures largely benefit from the perpetual marginalization of local communities, which results in the lack of sustainable change. The decimation of these power structures would require the removal of the privileges that global health in its current form provides foreign researchers.* [33]

In other words, decolonizing global health cannot be fully realized if incumbent powerholders do not lose out. Hirsch even argues that leveling the global health playing field means there will be a financial or emotional “cost” to institutions, as broken systems cannot be improved without taking out their broken parts [34]. Abimbola et al. suggest a “radical redistribution of funding away from HICs, a loss of epistemic and political authority, and a limitation to…intervene in LMICs” [15]. Delormier et al. call for the dismantling of vertical power structures in favour of “horizontal relational power structures” [35]. Meanwhile, Kunnuji et al. warn that a call for radical change, as opposed to incremental changes, would create more confusion about how to address decolonization, and leaves the movement “open to multiple interpretations with varied implications for global health practice” [17].

Decolonizing global health is a call for more equitable partnerships and a better power balance between the global north and global south [36]. Writing about the colonial legacy of malaria treatment, Bump and Aniebo express that:

*Decolonization is not fundamentally a rejection of knowledge accumulated under colonial arrangements, nor a return to pre-colonial conditions; instead it is a question of how we change objectives and accountabilities in favor of development and autonomy, and how we use that knowledge to move away from the production of inequality and dependency.* [37]

The machine of global health, rooted in colonialism, is structured such that the rich continue to accumulate wealth at the expense of the poor. In the context of decolonizing “big data” in global health, Qato calls for imperial powers and international aid agencies to redistribute their accumulated wealth from colonialism and exploitation, as well as from capitalizing on data accumulated from such exploited populations [38]. At the foundational level, Abimbola et al. point to education, particularly the continued development of quality research and teaching institutions in LMICs, to reduce dependence on HICs [15].

Evidently, global power asymmetries are structural. Decolonizing global health means to uproot these hegemonic structures that perpetuate dependence on HIC [15, 25]. Consequentially, global health might be unrecognizable upon its radical reformation into a new structure that upholds equity [15, 33].

#### Theme 2: Establishing agency and self-determination of the global south

As the global health arena continues to be dominated by money-wielding HIC funders [17, 23], there is a wide consensus among authors that ownership of health research and knowledge should be brought back to LMICs. Forsberg and Sundewall suggest that the global north must actively be inclusive to the global south in order to ensure “joint and equal ownership over research data” [36]. This active inclusivity means that HICs not only bow out of their positions of power, but also pull in more LMIC representatives on the vacated seats of the global health table. This is suggested by Abimbola and Pai:

*In research partnerships or funding decisions, it is not enough that HIC actors lean out. LMIC actors must also lean in— eg, by calling out parachute research, demanding reciprocity, setting up their own high-impact academic journals, or building high-quality schools of public health.* [39]

In other words, LMIC actors must be empowered to step into positions that HIC actors have long held. Only then can we see more local experts pitching inputs to propose local solutions for local problems. Counteracting parachute research would mean local authors communicating research findings to a local audience [40], and increasing primary authorship from LMIC researchers to highlight priorities and particular local research needs [41].

Authors believe that the time is ripe for enabling knowledge production from the global south. By elevating the voices and lived experiences of LMIC authors, Rees et al. envision that this will “provide a more equitable view of the way forward in just global health partnerships”, as well as seeking LMIC calls for how global health should be decolonized [42]. Furthermore, Büyüm et al. call for a bidirectionality of knowledge flows, and that the global south must drive discussions and practice [7].

Overall, LMICs must be given agency and be empowered to self-determine [39, 41], and there are three key areas for action in this space: (i) calls to decolonize global health should ideally come from the global south [7, 42], (ii) the global north must make space for those voices and actors from the south [22, 36, 39], and (iii) parachute research should be ushered out in favour of more equitable global health partnerships [36, 39].

#### Theme 3: Epistemic reformation and epistemic and ontological pluralism

As mentioned previously, Western paradigms of knowledge and ways of thinking have dominated the world. As such, colonial legacies have shaped ways of thinking. Abimbola et al. encourage the unlearning of the idea that Western knowledge systems are the only way to make progress in healthcare [15]. In a decolonized global health, it is envisioned that there is no such universalism, and that epistemic and ontological pluralism is the cornerstone of a broader epistemic reformation. Affun-Adegbulu and Adegbulu note that efforts in decolonizing global health must be rooted in dismantling the current concept of humanity—a concept arising from hegemonic Western universalism—which must be unlearned [43]. They further acknowledge that “there are many ways of being and doing,” therefore there is a need to actively engage with pluriversalities of being [43]. Jock et al. suggest that space needs to be created for different epistemological traditions [27]. Power and knowledge production are related [26]. Perkins et al. quote Lokugamage in disrupting the power imbalances in medical education, claiming that predominant biomedical approaches must yield to “medical pluralism” and Indigenous knowledge as alternatives [44]. Although there is a need to transform current predominant ontologies and epistemologies, Hindmarch and Hillier suggest that there is still a lack of “reconstructive discussion of alternatives to Western ontologies” [26].

To counteract hegemonic Western paradigms, global south epistemologies and ontologies should be given legitimacy [27]. A decolonized global health would mean undoing the dominance of Western ways of knowing or being [15], and promoting a pluralistic approach to knowledge as the vision going forward [43, 44].

#### Theme 4: Education

Current educational practices in global health and medical programs may carry and perpetuate some colonial legacies. As such, schools and institutions must be held accountable for being financially inaccessible to LMIC scholars [15], and for developing guidelines for responsible research conduct when collaborating with LMICs [45]. Virtual deliveries of education or remote learning may facilitate better inclusivity and diversity among succeeding generations of global health scholars [15]. Critical reform of educational curricula must then be facilitated in order to eliminate epidemiological biases and prejudices resulting from hegemonic Western practices [46]. Demir further mentions that institutions from the global south are not exempt from such biases [46]. These efforts would not be complete without directly addressing decolonial or anti-colonial practices. Curricula must educate students about colonial theory and its sociohistorical impacts on the developing world [47], critique structures that have been set up by colonial legacies, and build skills to dismantle such structures in an effort to redesign institutions and systems towards centering decoloniality [48].

Educational institutions are in a critical position to steer global health trajectory away from its colonial legacies. Curriculum reform [46–48] and responsible research guidelines [45] are some key ways in which education may contribute towards decolonizing global health.

#### Theme 5: Inclusivity, solidarity, and allyship

*“Effective allyship will require us to recognise the privileges, opportunities, resources, and power we have been accorded while others have been overtly or subtly denied them* [15].”

Decolonizing global health will be a collective effort. It requires privileges to be given up, which means that privileged HICs will need to cede their seats at the global health table to ensure participation from colleagues in LMICs. Hussain et al. call for HICs to “transfer power back to LMICs”, but this requires buy-in from the incumbent holders of power who are unwilling to cede their positions and allow for greater LMIC autonomy [22]. Authors suggest that the movement to decolonize global health is akin to recent progressive social justice movements. Khan et al. think that allies from the feminist movement are like-minded in seeking “system-wide change based on equity” [49], while Kunnuji et al. point to diversity, equity, and inclusion as a way to address the current lack of diversity in the boards of major global health institutions, building towards the elimination of “racial and supremacist ideas in global health governance” [17].

The decolonizing global health movement is seen as a unified effort and requires solidarity between the global north and south [15]. Social justice movements that call for rights and inclusivity for the marginalized are potential allies of this movement [17, 49].

### PART III—Who is participating in these discussions?

#### Theme 1: When was the decolonizing global health literature published?

With the exception of two, all articles included in this review were published in 2020 or later. Several entries were published in the wake of the George Floyd murder and the Black Lives Matter movement, as referenced in numerous articles [13, 27, 34, 50–52].

Meanwhile, around 58% (n = 53/92) articles mentioned the COVID-19 pandemic, on the heels of inequities mainly caused by the commodification of vaccines [18] resulting in “vaccine apartheid” [18, 47]. Much of the global south remained unvaccinated [101, 102 as cited in 53] while half of the world’s COVID-19 vaccine supply was kept by the global north [47].

Ultimately, the decolonizing global health literature gained traction as the COVID-19 pandemic brought long-standing global health inequities into sharper focus, further fuelled by broader conversations on social injustice after the killing of George Floyd.

#### Theme 2: Who is participating in these discussions per analyzed articles?

Several articles have established who the participants are in the decolonizing global health discussion. Rees et al. cite a study that found a lack of LMIC voices on the subject of decolonizing global health, that LMIC authors were not even aware of this movement [42]. Kunnuji et al. suggest that decolonization discourse is still a conversation only within HICs [17]. They further warn that calls to decolonize are unlikely to resonate with LMICs because outsiders cannot truly speak for what is best for LMICs [17]. Their analysis suggests that LMIC governments and actors must be convinced of the need to decolonize [17].

Naturally, authors express that the global south must facilitate decolonizing global health discourse [7, 32, 47].

#### Theme 3: Where are authors geographically based?

In analyzing which regions of the world authors were from, author affiliations were used as a proxy, and World Health Organization (WHO) regions were utilized to aid in the classification of countries. This analysis was conducted in two ways to provide a fulsome picture: (i) through solely assessing first authors’ affiliations and (ii) analyzing the affiliations of all authors. This dual analysis was conducted to both reflect where this work is being led from and who is being included. Additionally, author affiliation locations were classified according to the 2024 World Bank country income classifications, comprised of: high-income countries (HICs), upper-middle-income countries, lower-middle-income countries, and low-income countries (LICs) [103]. This additional analysis was conducted to better understand the variation in LMIC and HIC representation within each WHO region.

**Fig. 2:**
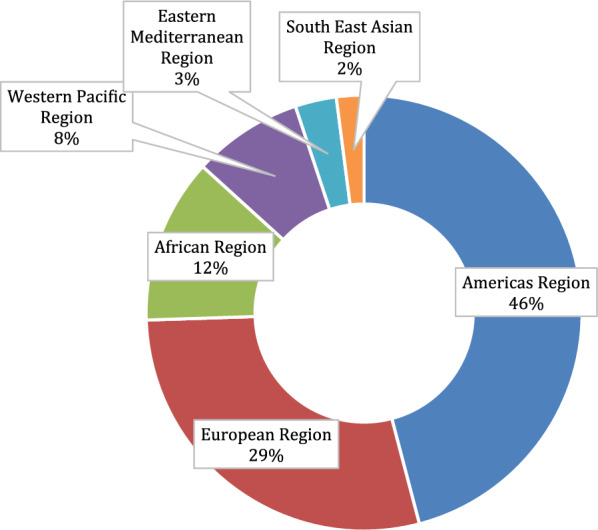
First author affiliations categorized by WHO region

When solely assessing first authors’ affiliations, a majority of first authors were from institutions in the global north. For the 91 included articles, there were a total of 101 first author affiliations, as some authors had multiple affiliations spanning different countries (see [37, 45, 50, 54–58]). Two authors had no affiliation listed, so these were omitted from the analysis, resulting in 99 first author institutions. Most first authors were situated in the Americas Region (n = 45/99; 46%), followed by the European Region (n = 29/99; 29%). These two regions accounted for almost 75% of all included articles. When narrowing in on authors based in the Western Pacific Region, this tally was dominated by authors based in institutions in Australia (n = 6), despite the region containing mostly LMICs. When combining all middle-income and low-income countries into one category as LMICs, authors based in LMICs accounted for only 22% (n = 22/99) of first author affiliations. The breakdown of first authors’ affiliation countries is available in Table 2, first authors’ affiliations categorized into WHO regions is available in Table 3, and a visualization of these regional differences is presented in Fig. 2.

**Table 2:** First authors’ affiliation countries

Country according to World Bank classification	Number of first authors
**High-income**	77
Australia	6
Belgium	5
Canada	6
Canada*	1
Chile	1
Norway	2
Puerto Rico	1
Sweden	1
Switzerland	2
United Kingdom	19
USA	33
**Upper-middle-income**	7
Botswana	1
Brazil	2
China	2
Costa Rica	1
South Africa	1
**Lower-middle-income**	11
Cameroon	1
India	2
Kenya	2
Nigeria	3
Pakistan	3
**Low-income**	4
Malawi	1
Rwanda	1
Uganda	2
Not applicable (affiliation not provided)	2
**Grand total**	101

*Delormier et al. represent the Kanien’kehá:ka nation, therefore we have categorized their work under Canada

**Table 3:** First authors’ affiliations categorized into WHO regions

WHO region	Number of first authors
African Region	12
Americas Region	45
Eastern Mediterranean Region	3
European Region	29
South East Asian Region	2
Western Pacific Region	8
Not applicable (affiliation not provided)	2
**Total**	101

When including all authors’ institutional affiliations, a total of 161 country affiliations were counted, as seen in Table 4. The breakdown of all authors’ affiliations by WHO regions is available in Table 5. One noteworthy finding is how the proportion of African Region countries increased from 12% (n = 12/99) as first author affiliations to just below 24% (n = 38/161) of all author affiliations. In numerous studies, the first author was affiliated with an American or European Region institution, but succeeding authors came from the African Region or American Region countries other than the United States or Canada. Only about 37% (n = 59/160) of authors had an affiliation based in a LMIC (please note that only 160 total affiliations were counted due to Palestine having no World Bank country classification [103]). Overall, the global north (Americas Region and European Region) accounted for 75% of first author affiliations, but dropped to around 63% for all author affiliations, as global-south-affiliated authors participated as non-first authors.

**Table 4:** All authors’ affiliation countries

Country according to World Bank classification	Number of authors
**High-income**	101
Australia	7
Belgium	5
Canada	9
Canada*	1
Chile	2
Germany	2
Ireland	1
Italy	2
Netherlands	1
Norway	2
Puerto Rico	1
Saudi Arabia	1
Spain	2
Sweden	2
Switzerland	5
Trinidad & Tobago	1
UAE	1
United Kingdom	20
USA	36
**Upper-middle-income**	18
Brazil	4
China	2
Costa Rica	1
Guatemala	1
Mexico	1
Peru	1
South Africa	8
**Lower-middle-income**	28
Bangladesh	1
Benin	1
Cameroon	2
Haiti	1
India	4
Kenya	5
Lebanon	1
Nigeria	5
Pakistan	2
Philippines	2
Senegal	1
Tanzania	2
Zimbabwe	1
**Low-income**	13
Ethiopia	1
Malawi	2
Mali	1
Rwanda	3
Sierra Leone	1
The Gambia	1
Uganda	4
**No classification**	3
Not applicable (affiliation not provided)	2
Palestine	1
**Grand total**	163

*Delormier et al. represent the Kanien’kehá:ka nation, therefore we have categorized their work under Canada

**Table 5:** All authors’ affiliations categorized into WHO regions

WHO region	Number of authors
African Region	38
Americas Region	59
Eastern Mediterranean Region	6
European Region	42
South East Asian Region	5
Western Pacific Region	11
Not applicable (affiliation not provided)	2
**Total**	163

## Discussion

This study illuminated the complex and often conflicting dogmas around what it means to decolonize global health. When analyzing how scholars understand “decolonizing global health”, its meaning was found to be rooted in three key components: (i) power asymmetries between the global north and south; (ii) a legacy of colonialism in global health or neocolonialism; and (iii) epistemic injustice. The second part of the analysis, which focused on how decolonizing global health can be acted on, demonstrated that decolonization of global health involves: (i) overhauling existing power structures; (ii) establishing agency and self-determination of the global south; (iii) epistemic reformation and epistemic and ontological pluralism; (iv) education; and (v) inclusivity, solidarity, and allyship. Taken collectively, these key components provide deep conceptual clarity which can promote critical reflection. This holistic collation of components over narrower definitions yields the strength that it has the potential to unveil aspects previously unconsidered and prompt reconsideration of assumed understandings. Reviewing the nuanced findings of this study and engaging in frank conversation can help ensure mutual understanding among stakeholders.

The findings show that discussions and definitions of decolonizing global health are often dominated by voices from and affiliated to the global north, as also determined in newly available work [10]. Most first authors were situated in the Americas Region (46%), followed by the European Region (29%), which collectively account for almost 75% of all included articles. This global north dominance perpetuates the same power imbalances that decolonization seeks to dismantle. This paradox highlights the need for better inclusion and steadier leadership within the global south to shape both the discourse and practice of decolonization. Two areas were outlined where attention can be paid in the short-term: promoting global south leadership in decolonization discourse, and advancing education and innovation in the epistemology of decolonization.

### Promoting global south leadership in decolonization discourse

The dominance of authors from the global north in this space suggests that to participate effectively in conversations around decolonizing global health, one often has to align—academically, professionally, culturally, and/or geographically—within the global north. In an effort to decolonize global health, it may seem paradoxical and somewhat ironic that countries of the global north, often the leading enactors of neocolonialism, should dictate the terms and structures of decolonial dialogues and interventions. This requirement only exacerbates the phenomenon of brain drain, where researchers and scholars from the global south leave their home regions for more resources, better opportunities, and greater visibility in the global north. This movement of talent perpetuates a cycle of knowledge marginalization, accentuating the imbalance of power dynamics between global south-based researchers and those now attached to more privileged regions who share the same perspectives and social and cultural conventions. Ultimately, this reliance on global north standards not only warps the narrative around decolonization by framing it predominantly from a global north perspective but also significantly reduces the leverage of individuals based in the global south to shape global health discourses.

In light of outlined imbalances, promoting global south leadership in decolonization discourse is essential. Echoing Abimbola [15], Guillaume [33], and Hirsch [34], the global north must cede its power to encourage the inclusion of the global south in these important decolonization conversations that are relevant to all. Should such a movement emerge from the south, it is essential that the south’s voices not only resound louder, but also, if necessary, prevail over those of the north. Concerted efforts must be prioritized to redress the balance and promote leadership of the global south in decolonization conversations that concern them.

Implementing practical strategies is necessary to catalyze this shift. It has become imperative to eliminate parachute research and eliminate any barriers that prevent individuals based in the global south from fully contributing to global dialogues (e.g., visas [104], travel). More than just empowering those in the global south, the aim is to recognize and promote leadership that exists across all regions, allowing voices from the global south to be heard and respected in the same way as those within the global north. Promoting global south leadership would reinforce the relevance and effectiveness of research solutions, responding appropriately to local and international health challenges. Further, the global north could greatly benefit from engaging with the global south and Indigenous populations, particularly around sustainable practices. Despite growing environmental threats, Indigenous peoples have defined the key determinants of planetary health, demonstrating that Indigenous knowledge systems provide an essential framework for preserving earth’s health [105].

### Advancing education and innovation in the epistemology of decolonization

Various authors have put forward dismantling the hegemony of Western universalism as the first step towards epistemic reform for decolonization to be effective and lasting [15, 28]. In the face of these epistemic injustices, many call for a pluralism of epistemologies and ontologies [15, 28, 44]. This implies an active approach to valuing the knowledge systems of not only the global south, but also Indigenous populations worldwide. Such pluralism needs to be actively embraced, which would mean embracing Indigenous knowledge and ultimately putting an end to its ongoing and systematic marginalization.

To truly transform systemic education on decolonization, voluntary changes in the framework governing the production and dissemination of knowledge are essential. Academic institutions have significant power in global health and can restructure existing curricula to reflect a more accurate and diverse reality of perspectives and knowledge. Addressing the legacy of colonialism and educating tomorrow’s practitioners, politicians, and researchers on the concepts of decolonization, can help steer global health towards a future that is more balanced and representative of its diverse realities.

However, the global community must anticipate resistance from the global north to strategies benefitting the global south. Krugman [5] addresses this theme of “elite capture”, which is a phenomenon where the decolonization agenda is co-opted and reconfigured by elites and powerful actors from the global north appropriate the dogmas, language, and concepts of decolonization to maintain their dominance over it. This strategy would contribute to erasing decolonization’s impact and hindering genuine change in its dynamics. It is crucial that efforts towards decolonizing have authentic impact and are not co-opted by existing actors in power who may resist fundamental shifts in the status quo.

### Limitations

The associated study protocol outlines three limitations that are byproducts of the intentional design of this study given the focus on characterizing mainstream discussions [1]. These three ‘limitations’ are that: only articles published in English were searched, grey literature was excluded, and texts falling outside of the parameters of global health were excluded. Each of these restrictions impacts the breadth of articles included in the study. However, the study was designed to focus on English-language articles in global health to bring clarity to mainstream discussions and help push debates. Future studies can cast a wide net to glean a fulsome understanding of the decolonizing literature beyond global health.

## Conclusions

Global health continues to be governed by a continuum of neocolonial principles, such as the control of intellectual property rights; the asymmetric distribution of resources; and the imposition of research priorities, access, and conditions by countries of the global north. The persistence of power disparities fostered by neocolonial attitudes and global north-centric actions pose a significant barrier to decolonizing global health. The movement to decolonize global health requires a collective commitment to transform the status quo—a commitment that heeds the risk of “elite capture”, overcomes entrenched interests in the global north, moves away from superficial reforms, engages with alternative epistemologies, and addresses the root causes of inequity embedded in colonial and actual power relations in place. The emergence of this topic presents an opportunity to establish consensus on a more concrete and unified definition and framework that guides actions, policies, and research initiatives. Ultimately, the global health community should seek to build a global health ecosystem that truly serves various needs and explores unconventional pathways that challenge the status quo.

## Data Availability

Data sharing is not applicable to this article as no datasets were generated or analyzed during the current study.

## Abbreviations

HICs: High-income countries
LMICs: Low- and middle-income countries
PRISMA-ScR: PRISMA guidelines for Scoping Reviews
WHO: World Health Organization

## Footnotes

1 We have intentionally elected to capitalize Western, as this “relat(es) to the countries of North America and Western Europe” [99]. Whereas we did not capitalize global south and north as these are not proper nouns.
